# AIDS patient with severe T cell depletion achieved control but not clearance of SARS‐CoV‐2 infection

**DOI:** 10.1002/eji.202149574

**Published:** 2021-12-04

**Authors:** Michele Spinicci, Alessio Mazzoni, Beatrice Borchi, Lucia Graziani, Marcello Mazzetti, Filippo Bartalesi, Annarita Botta, Marta Tilli, Filippo Pieralli, Marco Coppi, Nicla Giovacchini, Maria Grazia Colao, Riccardo Saccardi, Gian Maria Rossolini, Francesco Annunziato, Alessandro Bartoloni

**Affiliations:** ^1^ Department of Experimental and Clinical Medicine University of Florence Florence Italy; ^2^ Infectious and Tropical Diseases Unit Careggi University Hospital Florence Italy; ^3^ Intermediate Care Unit, Careggi University Hospital Florence Italy; ^4^ Microbiology and Virology Unit Careggi University Hospital Florence Italy; ^5^ Department of Cellular Therapies and Transfusion Medicine Careggi University Hospital Florence Italy; ^6^ Flow Cytometry Diagnostic Center and Immunotherapy (CDCI) Careggi University Hospital Florence Italy

**Keywords:** COVID‐19, HIV, CD4, immunocompromised, monoclonal, convalescent plasma

## Abstract

A late presenter AIDS patient with severe T cell depletion presented non‐severe COVID‐19 symptoms, with prolonged viral shedding. Our case report supports the hypothesis that an effective T cell response may be dispensable for the control of COVID‐19 progression to severe forms, while it may be necessary for SARS‐CoV‐2 clearance.

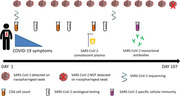

We report the case of a 26‐year‐old woman, with a clinical history of epilepsy since the age of eight, who was diagnosed with coronavirus disease 2019 (COVID‐19) by SARS‐CoV‐2 reverse‐transcriptase–polymerase‐chain‐reaction (RT‐PCR) on the nasopharyngeal swab (NS) (day 0), about one month after she had arrived in Italy from Morocco. The patient presented non‐severe COVID‐19 pneumonia, requiring admission at a local hospital. During the hospital stay, she received dexamethasone, low molecular weight heparin, and low‐flow oxygen support, showing clinical improvement. She was discharged on day 10, but two days later she was admitted again to the Emergency Room following seizure and loss of consciousness. A total body CT scan revealed multiple encephalic lesions and limited ground‐glass opacities of the left lung. The patient was transferred to the tertiary care Careggi University Hospital, Florence, Italy, where she was diagnosed with HIV infection. Real‐time PCR on cerebrospinal fluid was positive for *Toxoplasma gondii*, allowing the diagnosis of neurotoxoplasmosis in AIDS. Plasma HIV‐RNA load was 198,000 copies/mL with a CD4^+^ cell count of 2 × 10^6^/L (0.3%) and a CD8^+^ cell count of 344 × 10^6^/L (47.5%). *Pneumocystis jirovecii* infection was ruled out by PCR on bronchoalveolar lavage. Treatment with cotrimoxazole (10 mg/kg/day trimethoprim component in two divided doses) and tenofovir alafenamide/emtricitabine/bictegravir was introduced. The clinical course was complicated by seizure recurrence and opportunistic infections, including CMV reactivation, esophageal candidiasis – successfully treated with ganciclovir and fluconazole – and a cutaneous Kaposi's sarcoma on the right forearm. In the subsequent days CD4^+^ and CD8^+^ cell count slightly increased (6 × 10^6^/L [2.0%] and 229 × 10^6^/L [80.8%] on day 37, respectively; 30 × 10^6^/L [2.9%] and 757 × 10^6^/L [73.7%] on day 59, respectively), while on day 78 plasma HIV‐RNA load dropped to 113 copies/mL. During the hospital stay, the neurological condition gradually improved consistently with the reduction of the encephalic lesions at the CT‐scan control (day 66). Cotrimoxazole was switched to secondary suppression dosage after six weeks of treatment. The patient did not show any sign of respiratory involvement, nor did she present systemic symptoms related to SARS‐COV‐2 infection. However, NS specimens were persistently positive for SARS‐CoV‐2 by RT‐PCR (13 consecutive tests, between days 13 and 101).

On the basis of previous anecdotal reports on non‐HIV immunocompromised patients with persistent SARS‐CoV‐2 infection, two units of 200 ml high‐titers (1:320) COVID‐19 convalescent plasma were administered, 12 hours apart, on day 53 [1, 2, 3]. Given the persistent SARS‐COV‐2 positivity, on day 81 infusion of bamlanivimab/etesivimab 700mg/1400 mg was provided, without obtaining viral clearance, until day 107.

The nasopharyngeal samples taken on days 12 and 71 were subjected to whole‐genome sequencing by EasySeq RC‐PCR SARS‐CoV2 Whole Genome Sequencing kit (NimaGen BV, Nijmegen, NL) on an ILLUMINA MiSeq platform (San Diego, US), and the consensus sequences (available at www.gisaid.org, EPI_ISL_5010532 and EPI_ISL_5010531) were obtained by I‐Co‐Gen (Italian COVID‐19 Genomic) platform. The nearest neighboring GISAID sequence (EPI_ISL_728240), detected with the UCSC SARS‐CoV‐2 Genome Browser, was reported in Morocco at the end of December 2020 suggesting a possible foreign origin of the infection [4]. The sample EPI_ISL_5010531, taken on day 71, showed 8 points mutations compared to EPI_ISL_5010532, coding for amino acid substitutions in the ORF1a (V3718A), in the N‐protein (P13L and R185C), in the ORF9b (P10S), and in the S‐protein (T724D). Moreover, the Spike protein presented the Y144 deletion, which was also reported in variant of concern (VOC) Alpha and was demonstrated to abolish neutralization by a range of neutralizing antibodies [5]. On days 23 and 47 IgG antibodies against the SARS‐CoV‐2 Spike surface glycoprotein (S) and the nucleoprotein (N) were still undetectable by serological assays (LIAISON®SARS‐CoV‐2 S1/S2 IgG, DiaSorin Inc., USA; ARCHITECT®SARS‐CoV‐2 N IgG Immunoassay, Abbot, US). Twelve days after convalescent plasma infusion (day 65), SARS‐CoV‐2 IgG anti‐S were detectable (41 BAU/mL, with cut off 33.8 BAU/mL), while SARS‐CoV‐2 IgG anti N index was 0.83 S/CO (cut off 1.40 S/CO). Furthermore, on day 81 we evaluated cellular immunity to SARS‐CoV‐2. At this time point, CD4^+^ and CD8^+^ were still 1.1% and 84%, respectively, of total CD3^+^ T lymphocytes. Virus‐specific CD4^+^ T cells, identified by the production of IFN‐γ and IL‐2 following in vitro stimulation of PBMNC with peptide pools covering SARS‐CoV‐2 Nucleoprotein, Membrane, and Spike proteins were virtually absent, when compared to a representative COVID‐19 recovered individual. Regarding B cells, we found that the majority of circulating CD19^+^ cells displayed a naive phenotype, characterized by a CD27‐IgM^+^ phenotype, confirming that CD4^+^ T cell help is crucial to promote memory B cell formation. (Figure 1A and B). Accordingly, Spike‐specific B cells were not detected.

**Figure 1:**
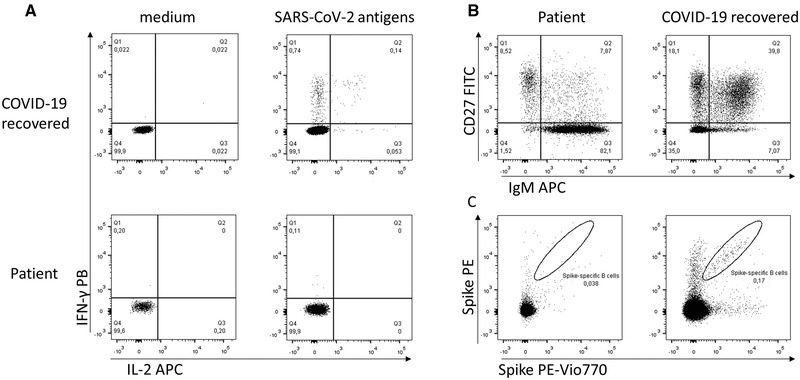
Identification of SARS‐CoV‐2 specific CD4^+^ T and CD19^+^ B cells. (A) Flow cytometric identification of SARS‐CoV‐2 specific CD4^+^ T cells. PBMNC from the patient and one COVID‐19 recovered subject were cultured for 6 h in vitro in medium alone or in presence of peptide pools covering Nucleoprotein, Membrane, and Spike proteins (SARS‐CoV‐2 antigens). SARS‐CoV‐2 specific CD4^+^ T cells were identified based on IFN‐γ and IL‐2 production. Plots are gated on live CD3^+^ CD4^+^ cells. (B) Phenotypic characterization of total CD19^+^ B lymphocytes from the patient and one COVID‐19 recovered subject. Naïve and memory B cell subsets were identified based on CD27 and IgM expression. Plots are gated on live CD19^+^ cells. (C) Flow cytometric identification of Spike‐specific B cells in the patient and one COVID‐19 recovered subject, performed with contemporary staining with recombinant Spike protein labeled with PE or PE‐Vio770. Plots are gated on live CD19^+^ cells.

The clinical features and outcomes of COVID‐19 among immunosuppressed patients need to be characterized further. In particular, current evidence does not establish whether immunosuppressed patients have more severe COVID‐19 outcomes or, conversely, they are protected from cytokine‐mediated detrimental inflammation and severe disease [6]. People living with HIV (PLH) have been reported to be a double risk for COVID‐19 adverse outcome, in comparison with HIV‐negative population [7]. Whether viral load and/or CD4^+^ cell count are associated with a higher risk of severe COVID‐19 and mortality have still to be established. The largest published cohorts reported an increase in crude mortality among PLH with a lower CD4^+^ cell count [8]. However, this association is probably driven by the high burden of comorbidities, which are more common in PLH than in uninfected people, and inversely proportional to CD4^+^ counts in PLH [9, 10]. In our case, the patient had a severe T cell depletion, as mirrored by multiple AIDS‐related opportunistic infections, but she was young and she had no meaningful comorbidities. Actually, COVID‐19 was pauci‐symptomatic, without any sign of evolution to acute respiratory distress syndrome or multi‐organ involvement, even in the absence of a specific antiviral treatment, thus supporting the hypothesis that concomitant conditions are more important than HIV‐associated immune dysfunction to predict COVID‐19 outcome in PLH.

On the other hand, the patient showed a prolonged SARS‐CoV‐2 shedding, as described in other immunosuppressed populations [11, 12]. Moreover, viral evolution leading to reduced sensitivity to neutralizing antibodies had been previously reported in an immunosuppressed individual with chronic SARS‐CoV‐2 infection treated with convalescent plasma [13]. Our case confirmed that in immunocompromised patients persistent infection and exposure to convalescent plasma are potential drivers for intra‐host viral evolution. In addition, treatments conferring passive humoral immunity against SARS‐CoV‐2 (convalescent plasma or monoclonal antibodies) failed to achieve viral clearance.

So far, T cells are considered to have a dominant role in the control and clearance of SARS‐CoV‐2 infection [14, 15]. Several cases of fully recovered SARS‐CoV‐2 infection in patients with genetic or pharmacological B cell depletion for the unrelated condition have been reported, suggesting that a protective immune response against SARS‐CoV‐2 can be produced even without humoral immunity [16, 17]. Based on our case report, an effective T cell response seems to be also dispensable for the control of COVID‐19 progression to severe forms, while it may be necessary for the full elimination of SARS‐CoV‐2.

## Conflicts of interest

The authors have declared no conflict of interest.

## Ethics and patient consent statement

The study was performed in accordance with the ethical principles of the Declaration of Helsinki and with the International Conference on Harmonization Good Clinical Practice guidelines. Patient's written consent was obtained.

### Peer review

The peer review history for this article is available at https://publons.com/publon/10.1002/eji.202149574.

## Data Availability

Data sharing not applicable to this article as no datasets were generated or analysed during the current study.
